# Signatures of resolution reprogramming in amyloid‐challenged microglia

**DOI:** 10.1002/alz.089644

**Published:** 2025-01-03

**Authors:** Galina Kondrikova, Eric D Hamlett, Dariusz Pytel

**Affiliations:** ^1^ Medical University of South Carolina, Charleston, SC USA; ^2^ MUSC, Charleston, SC USA

## Abstract

**Background:**

Specialized pro‐resolving mediators (SPMs) promote inflammatory resolution and homeostasis and are thought to have specific reprogramming effects on hman microglia. Decreased SPM levels have been correlated with chronic neuroinflammation, late‐stage Alzheimer’s disease (AD) and neuropathology in humans, yet few studies have explored the cellular signatures of resolution. Amyloid is though to bind one target resolution receptor, ChemR23, leading to internalization. In this study we explore if internalized ChemR23 receptor is part of a signaling endosome in microglial cells and how this interfaces with amyloid challenge and/or SPM mitigation.

**Method:**

We then challenged human microglial cell culture models (HMC3) with amyloid‐beta oligomer activation and/or administered RvE1 to promote resolution. We quantified resolution signaling responses using standard immunoblot. We measured microglial activation using immunofluorescence and cell characterization software. We examined the occurrence of ChemR23 receptor internalization and several endosomal markers by super‐resolution immunofluorescence microscopy.

**Result:**

RvE1 significantly reduces microglial activation and cytokine expression under amyloid challenge. We discovered that RvE1 leads to compensatory response in specific G protein coupled receptors within the resolution family 2) specific signaling events 3) unique endosome signatures, We are quantifying functionality of endosomes and other objectives to share at the poster at AAIC.

**Conclusion:**

We have demonstrated that RvE1 modulates inflammation in a mouse model and in a microglial cell line. Understanding the molecular signatures of resolution reprogramming in cells will be key to identifying prognostic indicators and molecular mechanisms of this unique understudied GPCR system and response in the brain.

